# GRA24-Based DNA Vaccine Prolongs Survival in Mice Challenged With a Virulent *Toxoplasma gondii* Strain

**DOI:** 10.3389/fimmu.2019.00418

**Published:** 2019-03-06

**Authors:** Bin Zheng, Di Lou, Jianzu Ding, Xunhui Zhuo, Haojie Ding, Qingming Kong, Shaohong Lu

**Affiliations:** ^1^Institute of Parasitic Diseases, Zhejiang Academy of Medical Sciences, Hangzhou, China; ^2^Zhejiang Provincial Institute of Parasitic Diseases, Hangzhou, China

**Keywords:** *Toxoplasma gondii*, immunization, DNA vaccine, GRA24, Th1/Th2 cytokines

## Abstract

*Toxoplasma gondii* causes infections in a wide range of intermediate hosts and remains a threatening disease worldwide because of the lack of effective drugs and vaccines. Dense granule protein 24 (GRA24) is a novel essential virulence factor that is transferred into the nucleus of host cells from the parasitophorous vacuole to regulate gene expression. In the present study, bioinformatic analysis showed that GRA24 had a high score for B-cell and T-cell epitopes compared with surface antigen 1 (SAG1), which has been studied as a promising vaccine candidate. As a DNA vaccine, pVAX1-GRA24 was injected intramuscularly into BALB/c mice and the induced immune response was evaluated. pVAX1-GRA24 induced high levels of a mixed Th1/Th2 cytokines at 6 weeks after immunization. Antibody determinations, cytokines [interferon gamma (IFN-γ), interleukin (IL)-12, IL-4, IL-10], antigen-specific lymphocyte proliferation, CD4+ and CD8+ T lymphocytes, and cytotoxic T lymphocyte activity showed that mice immunized with pVAX1-GRA24 produced specific humoral and cellular immune responses. The expression levels of interferon regulatory factor 8 (IRF8), nuclear factor kappa B (NF-κB), and T-Box 21 (T-bet) were significantly higher in the pVAX1-GRA24 immunization group than in the control groups. Survival times were prolonged significantly (24.6 ± 5.5 days) in the mice immunized with pVAX1-GRA24 compared with the mice in the control groups, which died within 7 days of *T. gondii* challenge (*p* < 0.05). The results of the present study showed that pVAX1-GRA24 induced a *T. gondii*-specific immune response and thus represents a promising candidate vaccine to treat toxoplasmosis.

## Introduction

*Toxoplasma gondii* belongs to the Apicomplexa phylum and can cause worldwide infections in a wide range of intermediate hosts, including humans, livestock, and marine mammals (1, 2). Studies indicate that approximately one-third of people worldwide are latently infected and carry *T. gondii* (3, 4). Toxoplasmosis is usually asymptomatic in people with normal immune function, but can cause serious complications and be life threatening in immunosuppressed populations, such as patients with HIV, patients with malignant tumors, and transplant recipients (5, 6). During pregnancy, the mother's first infection with *T. gondii* can lead to fetal abortion (7, 8). *T. gondii* infection also causes livestock death, resulting in heavy economic losses in veterinary and animal husbandry. In addition, humans can be infected via contact with infected animals (9). Interestingly, chronic toxoplasmosis is also associated with certain autoimmune diseases, ocular, mental, and neurodegenerative diseases (10–13).

Currently, the main measure to control *T. gondii* infection is the consumption of well-cooked meat and therapeutic drug treatment. However, these drugs are unsatisfactory because of their side effects. For example, they may cause teratogenic effects on the fetus and may not clear *T. gondii* cysts (14). Therefore, the discovery and development of safe and effective vaccines is preferred for the prevention and control of toxoplasmosis. Currently, there is a commercially licensed vaccine called Toxovax® (a live attenuated tachyzoite of the *T. gondii* S48 strain). The vaccine has side effects such as safety concern and inadequate efficacy and is only used in the veterinary industry in some countries (15, 16). In the past decade, significant progress has been made in the development of *T. gondii* vaccines, new antigens, adjuvants, and immunization strategies. Vaccine candidates range from attenuated vaccines, subunit vaccines, DNAs, RNAs, and exosomes (17, 18) The advantages and disadvantages of these vaccines have been studied primarily in mouse models to determine their potential to elicit cellular and humoral immune responses and their protective effects against *T. gondii* challenge (19–22). The main disadvantage of these vaccine models is that the great majority of them was not able to protect against the virulent challenge (23).

Current research on *T. gondii* vaccines has focused on finding novel vaccine candidates and evaluating their efficacy and immune strategies. Dense granule (DG) proteins (GRAs) have an important role in invasion, establishment of parasite vacuoles, manipulation of the host cell, and evading the host immune response (24). Among the GRAs, GRA24 is a newly identified protein that can be transferred into the nucleus of host cells from the parasitophorous vacuole to regulate gene expression. As a parasite-derived agonist, GRA24 can induce sustained p38α (also known as mitogen-activated protein kinase 14) autophosphorylation, without using the classical mitogen-activated protein kinase (MAPK) phosphorylation cascade, thereby enabling activation of transcription factors such as early growth response 1 (EGR1) or c-Fos (FOS, also known as Fos proto-oncogene, AP-1 transcription factor subunit) (25–27). Targeted deletion of *gra24* reduced the ability of mutant strains to cause disease in mice, which indicate that GRA24 is an essential virulence factor (28). Virulence factors have been regarded and verified as ideal materials to produce anti-*T. gondii* DNA vaccines, such as rhoptry protein (ROP) 5 and ROP18 (29, 30). However, it is unknown whether GRA24 could induce resistance to *T. gondii* infection in a mouse model.

In the present study, we investigated the immunogenicity and protective efficacy of the DNA vaccine pVAX1-GRA24 in the BALB/c mouse model, characterized the protective humoral and cellular immune responses, and discussed the potential immune mechanisms.

## Materials and Methods

### Epitope Prediction

DNASTAR software (Madison, WI, USA) was used to analyze the biochemical indexes of GRA24, including its antigenic index, hydrophilicity, flexible regions, and surface probability. The online service Immune Epitope Database (IEDB, http://tools.immuneepitope.org/mhcii/) was used to analyze the half maximal inhibitory concentration (IC50) values of peptides that bind to the MHC class II molecules of GRA24.

### Mice

Six-week-old female BALB/c mice were chosen as mouse models according the majority of previous studies on *T. gondii* vaccine (17) and purchased from the Zhejiang Experimental Animal Center in China and maintained under standard routine conditions. All procedures strictly abide by the legislation of the People's Republic of China on the use and care of laboratory animals.

### Parasites

The tachyzoites of *T. gondii* virulence RH strain (type I) were propagated and harvested, and then used to prepare soluble tachyzoite antigens (STAg), for mouse challenge experiments, and for total RNA extraction, as described previously (31).

### Preparation of Rabbit Anti-GRA24 Polyclonal Antibodies

Total RNA of *T. gondii* tachyzoites was extracted using the Trizol reagent (Invitrogen, Carlsbad, CA, USA). The cDNA was synthesized using a GoScript™ Reverse Transcription System (Promega, Madison, WI, USA). PCR was used to amplify the *gra24* gene (TGGT1_230180) of *T. gondii*, using the following primers designed by DNASTAR software (Madison, WI, USA): forward: 5'-CCGAAGCTTATGCTCCAGATGGCACGATA-3'; reverse: 5'- GCCCTCGAGTTAATTACCCTTAGTGGGTG−3'. The amplicon was cloned into the pET28a vector and identified by sequencing. The positive plasmid was designated as pET28a-GRA24. Recombinant GRA24 (rGRA24) was expressed in *Escherichia coli* BL21(DE3) and purified by affinity chromatography using Ni2^+^-NTA agarose columns (Qiagen, Hilden, Germany).

Purified rGRA24 was used to prepare rabbit anti-GRA24 polyclonal antibodies. In brief, two rabbits were immunized intravenously with purified rGRA24 protein (200 μg) to obtain a polyclonal antiserum. Each rabbit received three injections at biweekly intervals. Blood was collected from the ear vein of the rabbits 2 weeks after each biweekly injection.

### Construction and Expression of pVAX1- GRA24

The pVAX1-GRA24 plasmid constructed in the same manner as the above-described pET28a-GRA24 plasmid. The *gra24* gene was inserted into the eukaryotic vector pVAX1, confirmed by sequencing, and named pVAX1-GRA24. pVAX1-GRA24 was then transformed into *E. coli* DH5a. An Endofree plasmid giga kit (Qiagen, Chatsworth, CA, USA) was used to purify the pVAX1-GRA24 plasmid. The DNA concentration was determined by a NanoDrop 2000 instrument. Finally, pVAX1-GRA24 was diluted to 1 mg/ml by sterile endotoxin-free phosphate-buffered saline (PBS) and stored at −20°C before use.

The pVAX1-GRA24 expression plasmid was transfected to HEK293 cells (human embryonic kidney cells) using the lipo2000 reagent (Invitrogen, Carlsbad, CA, USA) according to the manufacturer's instructions. After 48 h, cell monolayers and supernatants were collected. pVAX1-GRA24 plasmid expression in the transfected cells was then detected by quantitative real-time PCR (qRT-PCR), western blotting and an indirect immunofluorescence assay (IFA).

### Indirect Immunofluorescence Assay

The cells transfected with pVAX1-GRA24 plasmid were fixed with 4% paraformaldehyde. After washing with PBS-0.1% Triton-X-100, rabbit anti-GRA24 polyclonal antibodies (1:100 dilution in PBS-0.1% Triton-X-100) were added to each well. Fluorescein isothiocyanate (FITC) labeled goat anti-rabbit IgG antibodies (diluted 1:2000; Catalog no., ab6721, Abcam, Cambridge, UK) were then added to detect the bound rabbit anti-GRA24 polyclonal antibodies. Finally, the specific fluorescence was imaged using a Nikon Ti-S fluorescence microscope (Nikon, Japan).

### Immunization and *T. gondii* Challenge

Four groups of female BALB/c mice (*n* = 25 per group) were immunized three times by intramuscular injection with 100 μl of diluted pVAX1-GRA24 (1 mg/ml), and boosted as an experimental group at intervals of 2 weeks. Mice injected with empty pVAX1 vector or PBS served as negative controls, and mice that were not injected served as blank controls. Blood from each group of mice was collected on days 13, 27, and 41, and serum was stored at −20°C.

Three weeks after the third immunization, 10 mice from each group were intraperitoneally challenged with 10^2^ tachyzoites of the *T. gondii* RH strain, and survival was recorded every day. All samples were detected in triplicate.

### Indirect ELISA

An indirect enzyme-linked immunosorbent assay (ELISA) was used to detect the titer of the prepared rabbit anti-*T. gondii* GRA24 polyclonal antibodies, and the total IgG and subtype antibodies IgG1, IgG2a after pVAX1-GRA24 immunization in mouse serum. Antigen concentration, and sera samples and cojugate dilutions were pre-acquired by checkerboard titration. Briefly, 96-well-plates were coated with 10 μg/ml of rGRA24 overnight at 4°C. The plates were washed three times with PBS containing 0.05% Tween-20 and blocked with 1% bovine serum albumin (BSA) for 2 h at room temperature. Sera were diluted in PBS, added to the wells, and incubated for 2 h. Plates were incubated with horseradish peroxidase (HRP)-conjugated goat anti-mouse/rabbit IgG, IgG1, or IgG2a (Abcam) for 2 h. After washing, immune complexes were revealed using tetramethylbenzidine as the substrate. Absorbance values at 450 nm were measured using an automatic ELISA reader (BioTek, Winooski, VT, USA). All assays were performed in triplicate.

### Lymphocyte Proliferation Assay

Briefly, the spleens of five mice from each group were aseptically removed 2 weeks after the last immunization. Spleen cells were harvested by pushing the spleen through a nylon sieve and then red blood cells were removed using red blood cell lysis buffer (Sigma, St. Louis, MO, USA). The purified spleen cells were resuspended in Dulbecco's modified Eagle's medium supplemented with 10% fetal bovine serum. Proliferation of spleen lymphocytes was detected using a CCK-8 kit (Dojindo, Kumamoto, Japan) according to the manufacturer's instructions. Purified spleen lymphocytes were added to 96-well-plates and when their density reached 2 ^*^ 10^5^, lymphocytes were stimulated with rGRA24 (10 μg/ml), Concanavalin A as a positive control (ConA; 5 μg/ml; Sigma) or Dulbecco's modified Eagle's medium alone as a negative control. Four days later, CCK-8 (Dojindo) was added to each well and incubated for 4 h to stimulate lymphocyte proliferation. The stimulation index (SI) was calculated using the following formula: stimulation index (SI) = (OD_570_ rGRA24/OD_570_ Control):(OD_570_ ConA/OD_570_ Control).

### Cytokines Detection

As in our previous study (30), cytokine assays were performed using commercially available kits (eBioscience, San Diego, CA, USA) according to the manufacturer's instructions. The culture supernatant of the splenic lymphocytes after re-stimulation of the rGRA24 was collected at different times. The levels of interleukin (IL)-4, IL-10, and interferon gamma (IFN- γ) and IL-12 were detected at 24, 72, and 96 h, respectively.

### Quantitative Real-Time PCR

Total RNA was isolated and first strand cDNA was synthesized as described in section Preparation of Rabbit Anti-GRA24 Polyclonal Antibodies. Quantitative real-time PCR was then performed on a CFX96 real-time PCR system (Bio-Rad, Hercules, CA, USA) using GoTaq® qPCR Master Mix (Promega). The *ACTB* gene (encoding β-actin) was used as an internal control to normalize the experimental results. The primers used are listed in Table 1.

**Table 1:** RT-PCR primers used to amplify the *NF-*κ*B p65, T-bet, IRF8*, and β*-actin* genes designed by DNASTAR software.

**Primer name**	**Sequence**
NF-κB p65-F	5′-GAACCAGGGTGTGTCCATGT-3′
NF-κB p65-R	5′-TCCGCAATGGAGGAGAAGTC-3′
T-bet-F	5′-GCCAGGGAACCGCTTATATG-3′
T-bet-R	5′-TGGAGAGACTGCAGGACGAT-3′
IRF8-F	5′-GCTGATCAAGGAACCTTGTG-3′
IRF8-R	5′- CAGGCCTGCACTGGGCTG−3′
β-Actin-F	5′-GCTTCTAGGCGGACTGTTAC-3′
β-Actin-R	5′-CCATGCCAATGTTGTCTCTT-3′

### Western Blotting Analysis

The expression of GRA24, T-Box 21 (T-bet), nuclear factor kappa B (NF-κB) p65 subunit, and interferon regulatory factor 8 (IRF8) were detected by western blotting, as described previously (30). The expression of β-actin in these cell lysates or the expression of H3 Histone in the cell nuclear lysates served as internal controls. Rabbit anti-GRA24 polyclonal antibodies were used as primary antibodies to detect GRA24. The other antibodies used in this study were purchased from Cell Signaling Technology, Inc. (Danvers, MA, USA) with the following catalog numbers: #8242 for NF-κB p65, #4499 for H3 Histone, and #5628 for IRF8. The antibodies were diluted 1:1000 to 1:2000 according to the manual. The secondary antibodies, including goat anti-rabbit IgG and goat anti-mouse IgG-horseradish peroxidase (HRP), were both diluted 1:5000 (Catalog no., ab6721 and ab6789; Abcam).

Transcription factors (T-bet, NF-κB p65 and IRF8) in the nucleus were detected by western blotting using the Nuclear and cytoplasmic Isolation Kit (Beyotime Institute of Biotechnology, Haimen, China).

### Flow Cytometry

Flow cytometry was used to detect the percentages of T cell subsets CD4+ and CD8+ in the splenocytes of mice in the pVAX1-GRA24, pVAX1, PBS, and blank groups. Splenocytes suspensions (5 ^*^ 10^5^ cells/ml) were dual-stained with anti-mouse CD3e-FITC+anti-mouse CD8-PE and anti-mouse CD3e-FITC+anti-mouse CD4-PE antibodies (eBioscience), for 30 min at 4°C in the dark. Cell populations were detected using a FACScan flow cytometer and analyzed using CellQuest software (BD Biosciences, Franklin Lakes, NJ, USA).

### Cytotoxic T Lymphocyte Activity Assays

Measurement of cytotoxic T lymphocyte (CTL) activity was performed using the CytoTox 96® Non-Radioactive Cytotoxicity Assay Kit (Promega, USA) according to the manufacturer's instructions. Briefly, spleen cells were cultured with 100 U/ml recombinant murine IL-12 (eBioscience) and used as effector cells. After 5 days, Sp2/0 mouse cells transfected with pVAX1-GRA24 were used as target cells. The effector cells were mixed with target cells at ratios of 10:1, 20:1, 40:1, and 80:1 and incubated for 6 h. The percentage of specific cell lysis was calculated as follows: (Experimental—Effector Spontaneous—Target Spontaneous)/(Target Maximum—Target Spontaneous) × 100.

### Statistical Analysis

GraphPad Prism 6.0 (GraphPad, San Diego, CA, USA) was used for the statistical analyses. Antibody levels, lymphocyte proliferation, and cytokine assays among the various groups were compared using an independent samples *t*-test. Mice survival times were analyzed using the Kaplan–Meier method. *p* < 0.05 were defined as statistically significant.

## Results

### Bioinformatic Analysis Identifies Good B Cell and T Cell epitopes in GRA24

**Figure 1:**
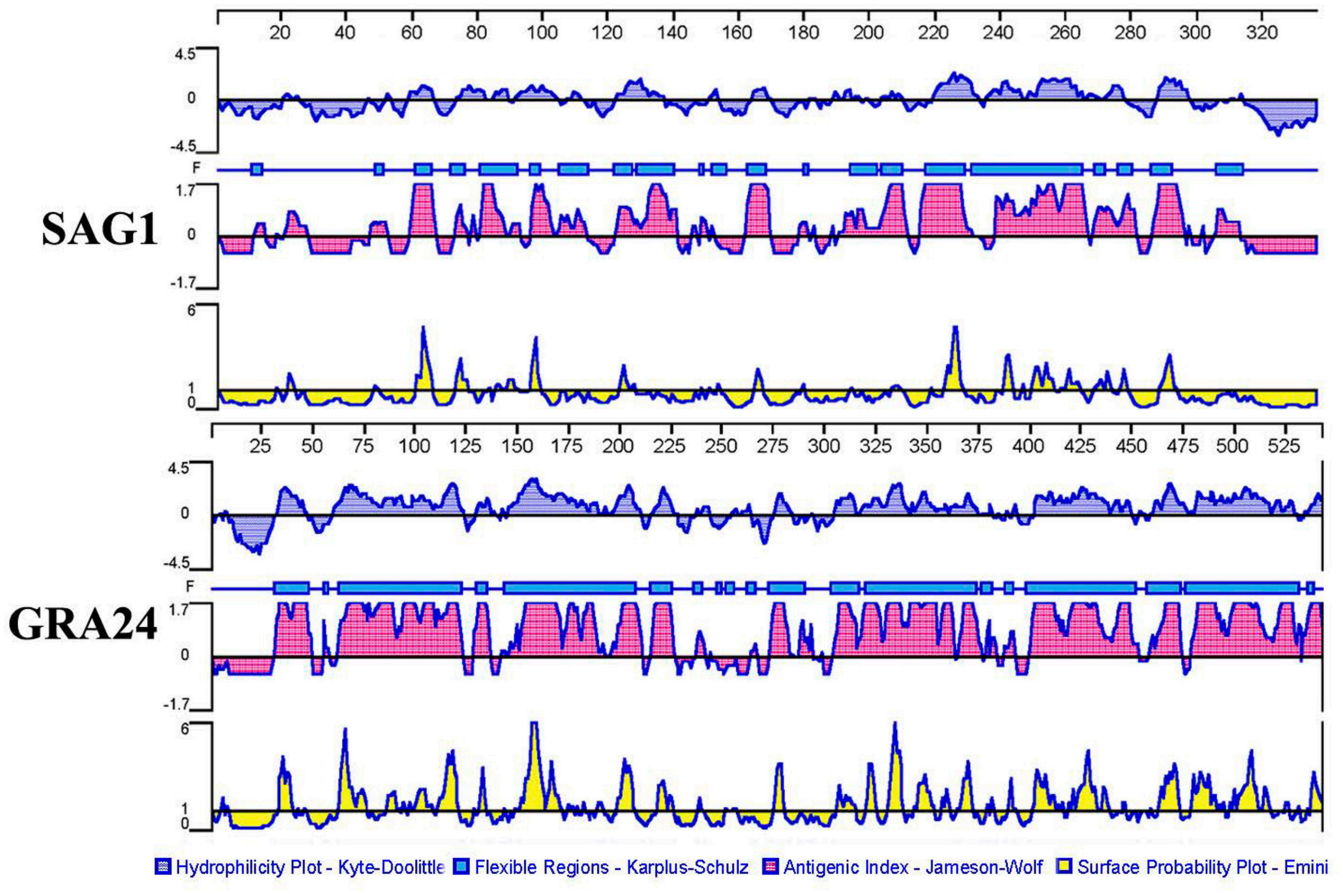
Plot of the DNASTAR-predicted hydrophilicity, flexible regions, antigenic index, and surface probability plot of the linear-B cell epitopes of GRA24 compared with those of SAG1.

According to Figure 1, GRA24 showed a higher score of surface probability, antigenic index, hydrophilicity, and flexible region than did SAG1 which was used as a reference because it is a promising DNA vaccine candidate that has induced effective cellular and humoral immune responses in immunized mice in previous studies (23).

In addition, we used the IEDB online service to analyze the T cell epitopes of GRA24 and SAG1. MHC class II molecules were associated with half maximal inhibitory concentration (IC50) values from the GRA24 peptides. The minimum percentile ranks of the GRA24 peptides are shown in Table 2. The IC50 values of HLA-DRB1^*^01:01, H2-IAb, and H2-IEd from GRA24 were lower than SAG1, which indicate that the GRA24 protein likely has strong binding to MHC-II.

**Table 2:** IC50 values for GRA24 and SAG1 binding to MHC class II molecules obtained using IEDB^a^.

**MHC II Allele^**b**^**	**Start-Stop**^**c**^		**Percentile Rank**^**d**^	
	**SAG1**	**GRA24**	**SAG1**	**GRA24**
H2-IAb	26–40	394–408	2.15	1.24
H2-IAd	21–35	3–17	0.34	3.93
H2-IEd	14–28	28–42	18.45	11.07
HLA-DRB 1^*^01:01	12–26	16–30	0.88	0.77

a *The Immune Epitope Database (http://tools.immuneepitope.org/mhcii)*.

b H2-IAb, H2-IAd, and H2-IEd alleles are mouse MHC class II molecules; the HLA-DRB1^*^01:01 allele is a human MHC class II molecule.

c *15 amino acids were chosen for analysis*.

d *Low percentile indicates high level binding according to the software instructions*.

Taken together, the bioinformatic analyses showed that the peptides of GRA24 had a higher score of linear-B cell epitopes and lower percentile of IC50 values than SAG1.

### Expression of pET28a-GRA24 in *E. coli* BL21(DE3) and pVAX1- GRA24 in HEK293 cell

**Figure 2:**
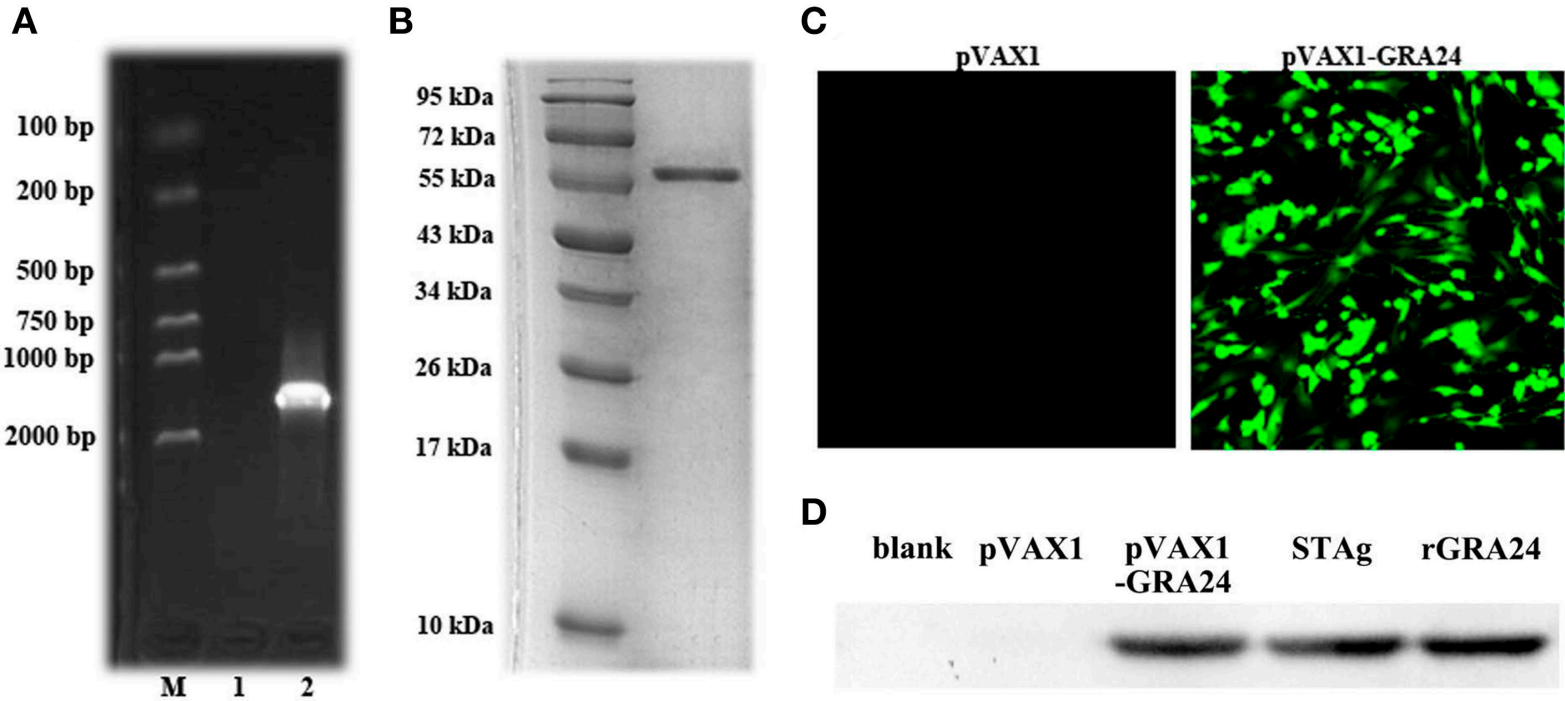
Expression of pET-28a- GRA24 in *E. coli* BL21(DE3) and pVAX1- GRA24 in HEK293 cell. **(A)** PCR amplified *gra24* gene detected by 1.5% agarose gel. M: Markers; 1: Blank control; 2: coding region (CDS) of *T. gondii*. **(B)** Analysis of purified rGRA24 by SDS-PAGE. **(C)** The expression of pVAX1-GRA24 in HEK293 cells by an indirect immunofluorescence assay. **(D)** Western blotting detection of the expression of GRA24 in HEK293 cell lysates and STAg. Blank: non-transfected cells; pVAX1: empty pVAX1 transfected cells; pVAX1-GRA24 transfected cells; soluble antigen of *T. gondii*; rGRA24 acts as a positive control.

As shown in Figure 2A, we amplified the *gra24* gene from *T. gondii* cDNA, which is 1,629 bp in size and is predicted to encode a GRA24 protein of ~57.4 kDa. We constructed the prokaryotic expression vector pET28a-GRA24 and transformed it into *E. coli* BL21(DE3). After induction and purification, we obtained highly pure rGRA24 using SDS-PAGE analysis (Figure 2B). A rabbit polyclonal antibody prepared using rGRA24 was able to specifically recognize the native GRA24 in a *T. gondii* STAg preparation (Figure 2D).

We transfected the constructed pVAX1-GRA24 eukaryotic expression vector into HEK293 cells. Using indirect immunofluorescence, we detected green fluorescence in cells transfected with pVAX1-GRA24, whereas no green fluorescence was detected in cells transfected with the empty vector pVAX1 (Figure 2C). At the same time, we detected cell lysates by western blotting using the rabbit anti-GRA24 polyclonal antibodies, and detected a single specific GRA24 band in cells transfected with pVAX1-GRA24. No band was detected in the control groups (Figure 2D). These results indicated that GRA24 could be expressed from pVAX1-GRA24 in eukaryotic cells.

### pVAX1-GRA24 Immunized Mice Produce High Levels of IgG and Subtype IgG1, IgG2a Antibodies

**Figure 3:**
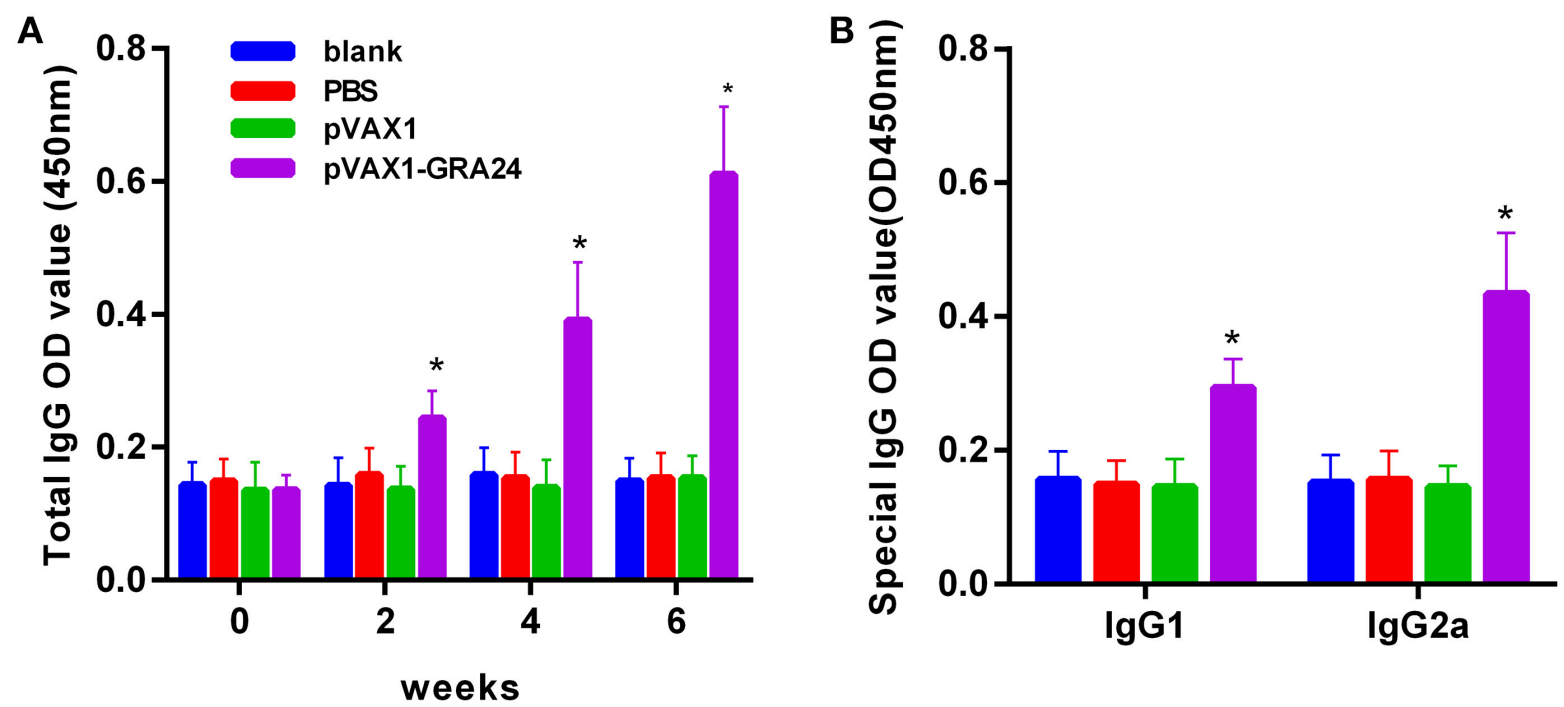
DNA vaccination with pVAX1-GRA24 induced dynamic changes in humoral responses in BALB/c mice. **(A)** Total IgG; **(B)** IgG1, IgG2a. The OD450 value of total IgG were detected on 0, 2, 4, and 6 weeks post-vaccination. The OD450 value of IgG subclass (IgG1, IgG2a) were detected at 6 weeks post-vaccination. Data are expressed as mean ± SD (*n* = 20) from three independent experiments. Asterisks (^*^) indicate statistically significant differences (*p* < 0.05) compared with the control groups.

To assess the changes in antibody levels caused by three consecutive inoculations of the pVAX1-GRA24 DNA vaccine, the total IgG antibody titer after each injection, and the distribution of IgGl and IgG2a isotypes 2 weeks after the final injection were tested. Serum total IgG levels were significantly higher in animals injected with pVAX1-GRA24 than in the control groups (*p* < 0.05). In addition, the OD value of IgG increased with subsequent injection. None of the control groups showed statistically significant levels of IgG compared with that in the immunized groups (*p* > 0.05, Figure 3A). High levels of IgG1 and IgG2a were detected in animals injected with pVAX1-GRA24 compared with those in the control group (*p* < 0.05; Figure 3B), wherein the IgG2a level was significantly higher than the IgG1 level, indicating that the pVAX1-GRA24 vaccine induces a dominant Th1-type cellular immune response.

### Lymphocyte Proliferation Ability Increased in pVAX1-GRA24 Immunized Mice

The lymphocyte proliferative response was determined using rGRA24 as a stimulator, ConA (positive control) and medium only (negative control). As shown in Table 3, the level of spleen cell proliferation in the mouse group injected with pVAX1-GRA24 was increased compared with those in the three control groups (SI: 2.19 ± 0.13; *p* < 0.05). In addition, there was no statistically significant difference in the spleen lymphocyte proliferation index among the three control groups (*p* > 0.05).

**Table 3:** Cytokine production and the proliferative responses of splenocytes from BALB/c mice immunized with PBS, pVAX1, pVAX1-GRA24, or blank control.

**Groups (*n* = 5)**	**Cytokine production (pg/ml)**				**Proliferation (SI)^**a**^**
	**IFN-γ**	**IL-12**	**IL-4**	**IL-10**	
Blank control	39.56 ± 4.86	35.29 ± 4.21	43.21 ± 3.69	25.69 ± 2.39	0.95 ± 0.13
PBS	37.59 ± 3.98	39.15 ± 4.26	39.65 ± 4.12	27.19 ± 3.78	1.03 ± 0.14
pVAX1	39.74 ± 4.56	42.11 ± 3.98	41.59 ± 3.59	28.13 ± 4.12	0.99 ± 0.17
pVAX1-GRA24	535.59 ± 59.78^*^	325.18 ± 37.59^*^	223.89 ± 30.19^*^	201.23 ± 29.15^*^	2.19 ± 0.13^*^

a *SI, stimulation index*.

* *p < 0.05 compared with the control groups*.

### Th1 Cytokines (IFN-γ, IL-12) and Th2 Cytokines (IL-10, IL-4) Increased Levels in pVAX1-GRA24 Immunized Mice

To determine the type of the T helper cell response, we used ELISA to measure Th1- and Th2-type cytokines released in culture supernatants after rGRA24 re-stimulation of spleen cells. As shown in Table 3, spleen cells of mice immunized with pVAX1-GRA24 secreted a large amount of IFN-γ, IL-12, IL-4, and IL-10. Significantly high levels of Th1 type cytokines (IFN-γ: 535.59 ± 59.78 pg/ml; IL-12: 325.18 ± 37.59) were observed in splenocyte cultures of mice immunized with pVAX1-GRA24 compared with that of the control mice (*p* < 0.05). By contrast, significantly high levels of Th2 type cytokines (IL-4: 223.89 ± 30.19 pg/ml; IL-12: 201.23 ± 29.15) were also observed in splenocyte cultures of pVAX1-GRA24-immunized mice, compared with mice immunized with pVAX1, PBS, or blank control (*p* < 0.05).

### pVAX1-GRA24 Immunization Increased CD4+ and CD8+ T Cells Cell Levels

**Figure 4:**
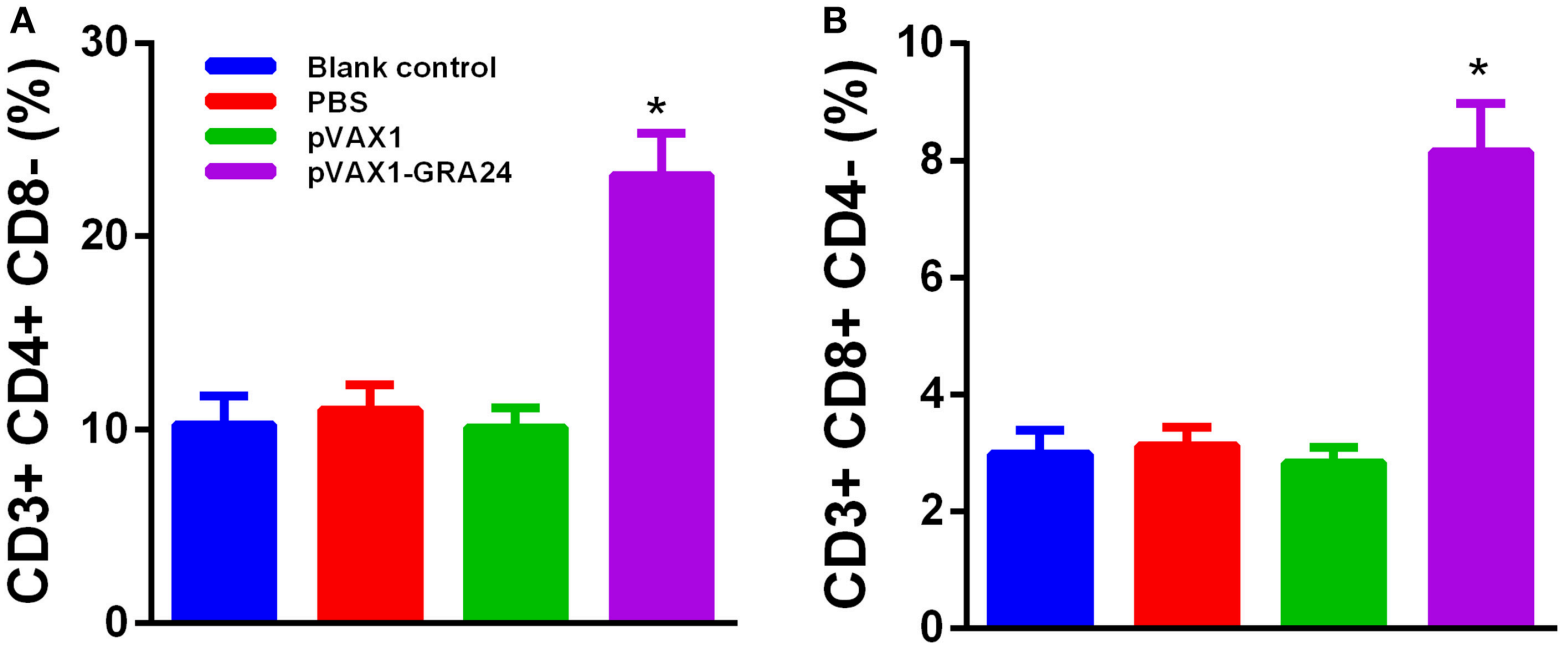
Percentages of CD4+ T cells and CD8+ T cells subsets in immunized BALB/c mice. **(A)** CD4+ T cells; **(B)** CD8+ T cells. Results are shown as the mean ± SD (*n* = 5 mice in each group) from three independent experiments. ^*^p < 0.05 compared with the controls. PBS, phosphate-buffered saline.

To determine whether pVAX1-GRA24 vaccination activated CD4+ or CD8+ T cells, flow cytometry was used to determine the percentage of CD4+ and CD8+ T lymphocytes in the spleens of mice from each group. Figure 4 shows that the percentage of CD8+ and CD4+ T cells in the mice immunized with pVAX1-GRA24 increased significantly compared with that in the control groups (*p* < 0.05). The differences in the percentage of CD8+ and CD4+ T cells among the control groups were not significant (*p* > 0.05).

### The Expression of Cytokine-Related Transcription Factors Increased After pVAX1-GRA24 Immunization

**Figure 5:**
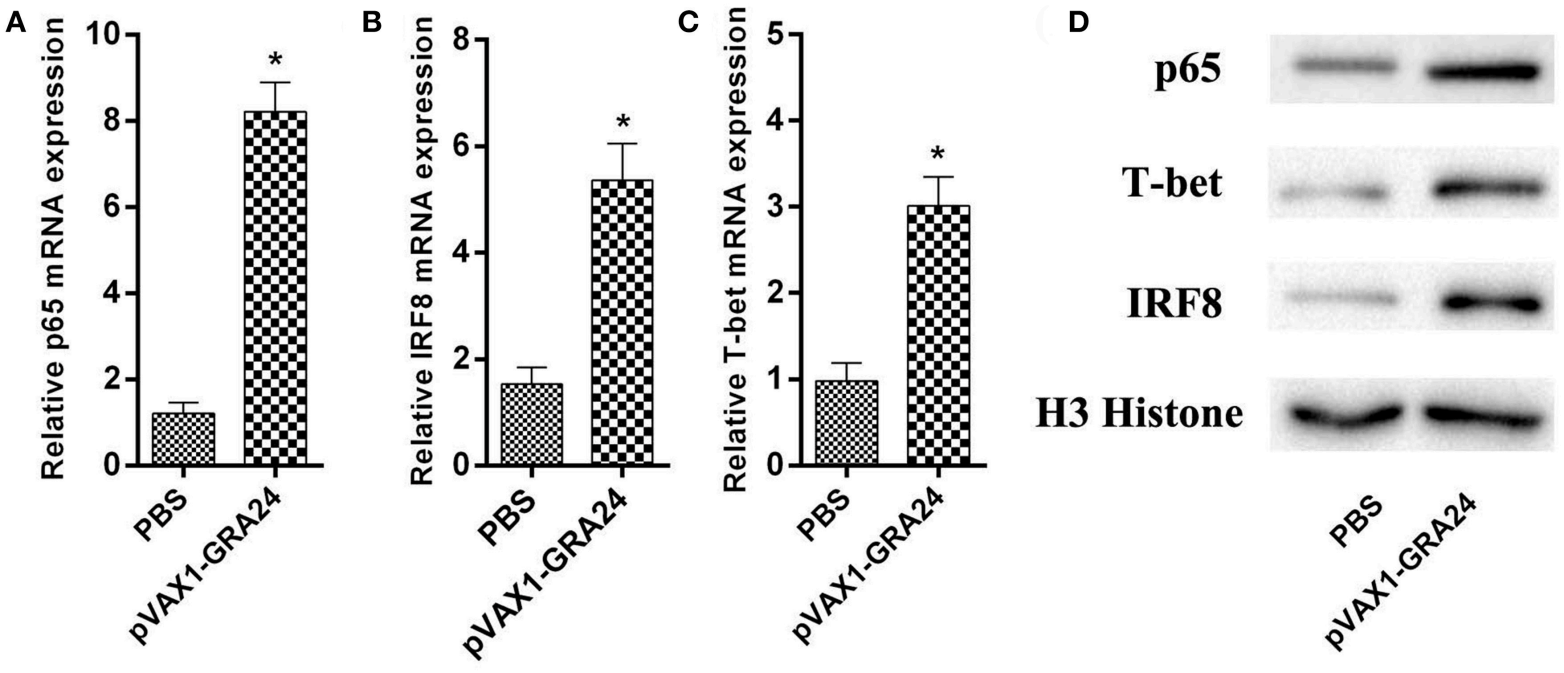
The mRNA and protein expression levels of p65, IRF8, and T-bet. **(A–C)** The mRNA levels of *p65, IRF8*, and *T-bet*; **(D)** The protein levels of p65, IRF8 and T-bet. Data are expressed as the mean ± SD (*n* = 5) from three independent experiments. Asterisks (^*^) indicate statistically significant differences (*p* < 0.05) compared with the control groups. PBS, phosphate-buffered saline.

We detected the mRNA and protein expression levels of the transcription factors p65, IRF8, and T-bet, respectively, using qRT-PCR and western blotting, respectively. The difference in expression of these three genes/proteins between control mice and pVAX1-GRA24 immunized mice was analyzed. The results showed that the expression levels of p65, IRF8, and T-bet were significantly higher in the pVAX1-GRA24 immunization group than in the control groups (*p* < 0.05, Figure 5). There was no significant difference in the expression levels of these three transcription factors among the three control groups (*p* > 0.05). This result indicated that pVAX1-GRA24 immunization induced increased expression of p65, IRF8 and T-bet.

### High CTL Activity Detected in Spleen Cells of Mice Immunized by pVAX1-GRA24

**Figure 6:**
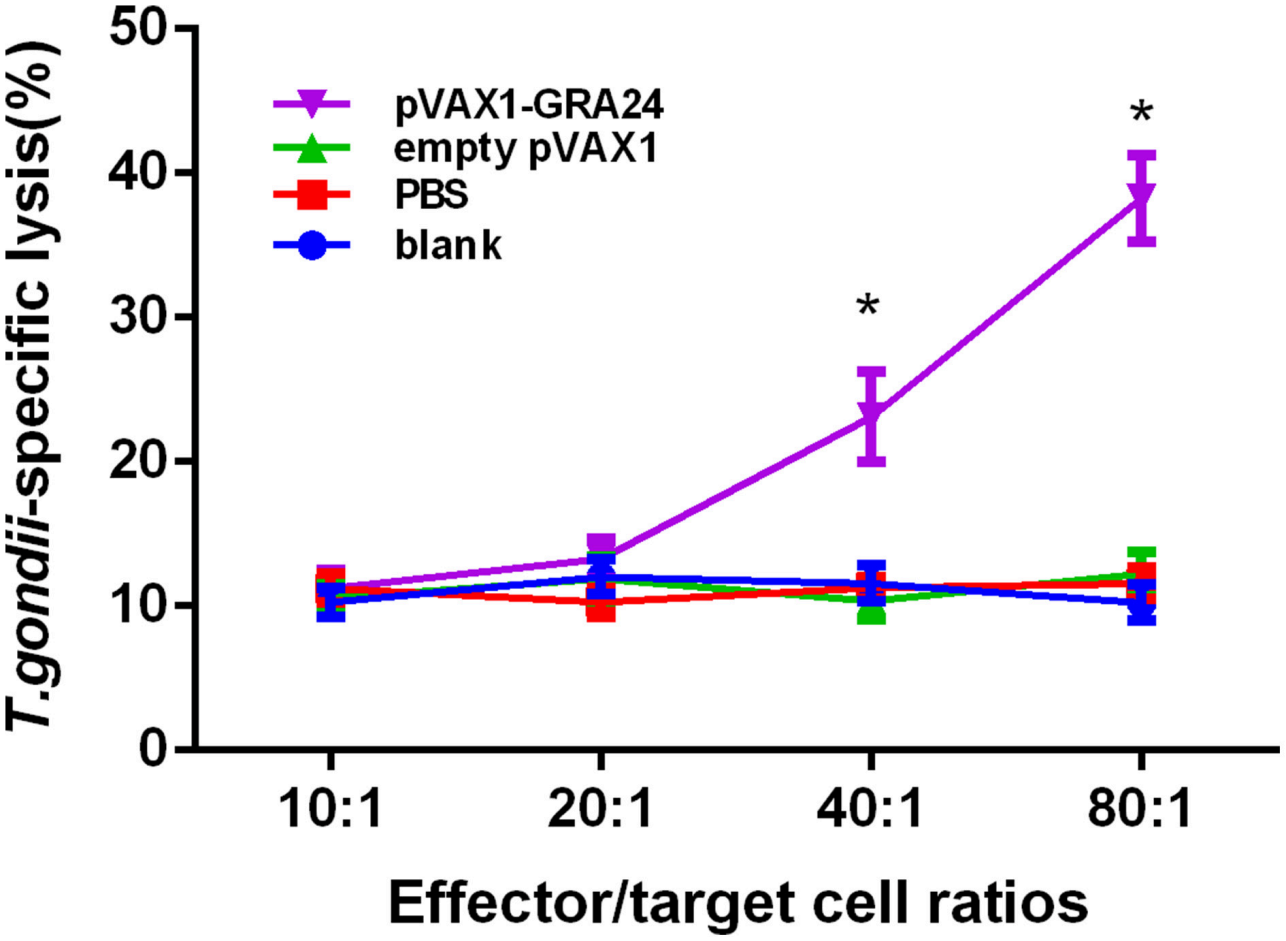
CTL activities of spleen lymphocytes in pVAX1-GRA24 immunized mice. The vertical axis shows *T. gondii-*specific lysis as a percentage of the total possible lysis (%). CTL, cytotoxic T lymphocyte; PBS, phosphate-buffered saline. Asterisks (*) indicate statistically significant differences (*p* < 0.05) compared with the control groups.

Efficient protection against *T. gondii* is critically dependent on pathogen-specific cytotoxic T lymphocyte (CTL) responses. We analyzed the CTL activity of spleen lymphocytes in mice immunized with pVAX1-GRA24 and control mice. As shown in Figure 6, with the gradual increase in the ratio of effector cells to target cells, the CTL activity of spleen cells of mice immunized with pVAX1-GRA24 also gradually increased. When the ratio of effector cells to target cells was 40:1, there was a significant difference compared with the three control groups (*p* < 0.05), and at 80:1, the CTL activity reached its highest level (*p* < 0.05). There was no significant difference in CTL activity of spleen lymphocytes among the three control groups (*p* > 0.05).

### Prolonged Survival Time of Mice Immunized by pVAX1-GRA24

**Figure 7:**
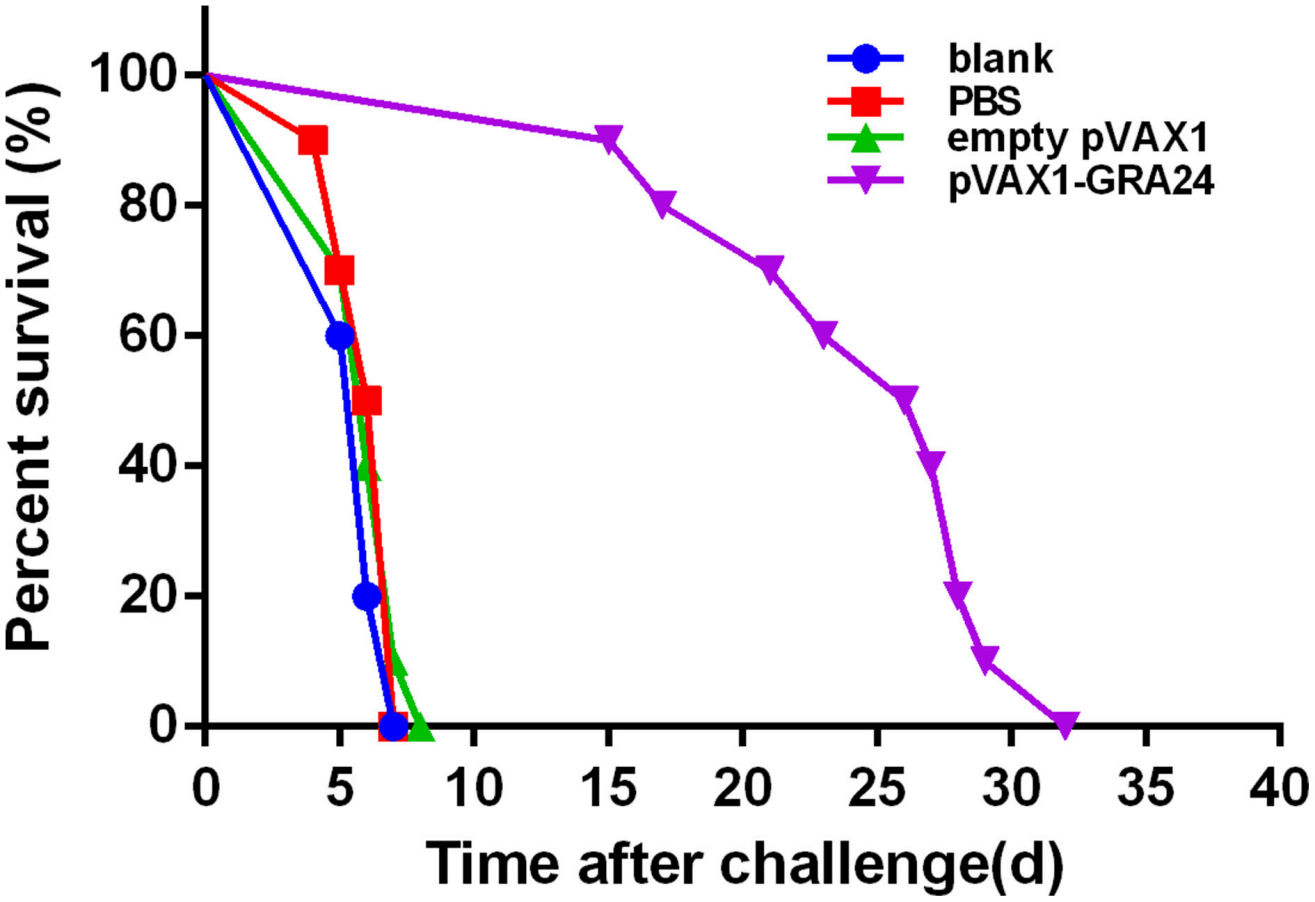
Survival rate of mice immunized with pVAX1-GRA24, pVAX1, PBS, and untreated mice. Each group comprised 10 mice. PBS, phosphate-buffered saline.

To assess the protection provided by the DNA vaccines, all experimental mice were challenged with tachyzoites of the *T. gondii* RH strain. As shown in the survival curve (Figure 7), after challenge with the virulent *T. gondii* RH strain, compared to mice receiving pVAX1, PBS, or a blank control (all of these mice died within 7 days, *p* > 0.05), pVAX1-GRA24 immunized mice showed prolonged survival time (24.6 ± 5.5 days, *p* < 0.05).

## Discussion

*T. gondii* is an obligate intracellular Apicomplexan protozoan with worldwide distribution and major medical and veterinary importance. *T. gondii* can infect humans and other warm-blooded animals, causing toxoplasmosis, which is a serious worldwide public health problem. Currently, there is no effective and side-effect-free therapeutic drug for the treatment of toxoplasmosis. Therefore, the development of a safe and effective *T. gondii* vaccine is urgently required. In recent years, with the identification of new antigens, adjuvants, and immune strategies, the development of *T. gondii* vaccines has progressed significantly. Vaccine candidates range from recombinant antigens to synthetic peptides, naked DNA or RNA, and microparticles. The advantages of these vaccines have been studied in animal models to assess their potential to elicit cellular and humoral immune responses and their protective effects against *T. gondii* attack. Previous studies have shown that DNA vaccines are a promising method to protect animals against *T. gondii* infection because of their low production cost, thermal stability, and their ability to induce cellular and humoral immune responses (23). In this study, we selected plasmid pVAX1^TM^, which meets the guidelines for design DNA vaccines of U.S. Food and Drug Administration (FDA) (32). We investigated the possibility of a novel *T. gondii* virulence-associated factor GRA24 as a DNA vaccine molecule to prevent mice toxoplasmosis. The results showed that pVAX1-GRA24-immunized mice developed humoral and cellular immunity against *T. gondii*, prolonging the survival time of mice challenged with virulent *T. gondii*.

Adaptive immunity is mediated by T- and B-cells, which are immune cells capable of developing pathogen-specific memory that confers immunological protection. Memory and effector functions of B- and T-cells are predicated on the recognition through specialized receptors of antigens in pathogens. More specifically, B- and T-cells recognize portions within their cognate antigens known as epitopes. Identifying epitopes in antigens has important roles in many aspects, such as elucidating disease etiology, immune monitoring, developing diagnostic assays, and designing epitope-based vaccines (33). Bioinformatics has been widely used to predict gene structures, functions, and epitopes. The epitopes of many molecules in studies of *T. gondii* DNA vaccines have been predicted by bioinformatics such as SAG4 (34), ROP21 (35), TgDOC2C (36), etc. In the present study, the bioinformatic analyses showed that the peptides of GRA24 had a higher score of linear-B cell epitopes and lower percentile of IC50 values than SAG1, which indicated a promising prospect of vaccine production.

Humoral immunity plays a very important role in resistance of *T. gondii* infection. Antibodies can regulate parasite phagocytosis, prevent invasion, and stimulate the classical complement pathway to provide effective protection (37). In this study, we found high levels of specific anti-*T. gondii* IgG in the serum of pVAX1-GRA24 immunized mice. A large number of studies have confirmed that the Th1 type immune response plays an important role in effectively resisting *T. gondii* infection under natural conditions (38). Our results showed that pVAX1-GRA24 immunization could induce high levels of IgG1 and IgG2a antibodies. The level of IgG2a antibodies was significantly higher than that of IgG1, indicating that pVAX1-GRA24 induces a mixed Th1/Th2, but Th1-dominated, immune response. These results are similar to those of other previously reported *T. gondii* DNA vaccines (31, 34, 35). The results for the antibody subtypes was confirmed by cytokine detection in mouse spleen cell culture supernatants. In pVAX1-GRA24-immunized mice, we detected high levels of Th1 (IFN-γ, IL-12) and Th2 (IL-4, IL-10) cytokines.

High levels of the Th1 type cytokine immune response play a crucial role in host resistance to *T. gondii* infection. Initiation of IL-12 production in host immune cell is essential for host resistance to *T. gondii* (39). Blocking IL-12 or knockout of the subunit of IL-12 (IL-12p40 or IL-12p35) resulted in the development of acute susceptibility to *T. gondii*, which is similar to that observed in MYD88 deficient mice. In addition, IL-12 is essential for the production of IFN-γ in the acute and chronic phase of infection (40). IFN-γ is the major effector molecule required for host resistance to parasites. At least three IFN-γ-mediated protective mechanisms have been identified. First, IFN-γ inhibits the growth of *T. gondii* by inducing tryptophan degradation (41). Second, IFN-γ can mediate the biochemical pathway of nitrogen oxides (NO) to clear *T. gondii* and degrade L-arginine, which is necessary for *T. gondii* survival (42). Third, reactive oxygen intermediates are associated with cell type-specific and species-specific IFN-γ-mediated elimination of *T. gondii* (43). Fourth, the immune-related GTPases (IRGs) are a family of IFN-γ-inducible proteins that are indispensable for host resistance to *T. gondii* (44). IRGs accumulate on parasitophorous vacuole, resulting in destruction of vacuoles and elimination of parasites by lysosomal mediated degradation (45). In the present study, spleen cells of mice were re-stimulated with rGRA24 *in vitro*, which induced high levels of Th1-type cytokines (IFN-γ, IL-12) in pVAX1-GRA24 immunized mice. In addition, we also detected high levels of Th2 type cytokines (IL-4, IL-10). This result was similar to the results of our previous study of the *T. gondii* rROP5 subunit vaccine (30). IL-4 functions to enhance IFN-γ production in the late stage of infection. In IL-4^−/−^ mice, their impaired ability to produce IFN-γ likely contributes to their increased susceptibility to severe toxoplasmic encephalitis (46). However, IL-10 has a central role in limiting inflammation and inhibiting CD4^+^ T cell-mediated severe immunopathology, which is associated with increased pro-inflammatory cytokine production during *T. gondii* infection (45). Pregnant mice immunized with *T. gondii* ΔGRA17 mutant were able to produce high levels of IL-10, which could modulate the pathological damage in fetal mice induced by a large number of Th1 cytokine IFN-γ induced by *T. gondii* challenge (47). Maintaining immune homeostasis when suffering from toxoplasmosis requires the ability to clear pathogens and control host immune responses to prevent severe immunopathology.

IL-12 is important for the production of IFN-γ, which in turn induces differentiation of Th1 T lymphocytes and CD8+ and NK cells, thereby controlling *T. gondii* infection. IRF8 is an essential transcription factor regulating the expression of IL-12p40 and IL-12p35, downstream of TLR11 and MYD88 activation (48). The results indicate that when IRF8-deficient mice are challenged with *T. gondii*, they are unable to produce IL-12 or IFN- γ, and they die rapidly from infection, despite having normal levels of tumor necrosis factor (TNF), IL-6, IL-1α and IL-1β65 (49). In the present study, we found that the expression of IRF8 was significantly increased in spleen lymphocytes of pVAX1-GRA24-immunized mice. This indicated that pVAX1-GRA24 immunization can induce IL-12 expression via the IRF8 pathway. Similar to the IRF8 signal pathway, the NF-κB-mediated signal transduction pathway also plays an important role in the production of IL-12 or IFN-γ. We found that the expression of NF-κB was significantly increased in spleen lymphocytes of pVAX1-GRA24-immunized mice. This indicated that pVAX1-GRA24 immunization could induce IL-12 or IFN-γ expression via the NF-κB pathway. T-bet specifically regulates Th0 differentiation, plays a key role in the Th1/Th2 switch, and is selectively expressed in Th1 cells. In the present study, pVAX1-GRA24 immunization induced T-bet expression, which was consistent with the detection of cytokines and IgG subtype antibodies. This finding suggests that increased IFN-γ production may be caused by activation of the IRF8 pathway, NF-κB pathway, and T-bet-mediated activation of CD4+ T cells and natural killer (NK) cells (50).

IFN-γ-dependent host protection against *T. gondii* is mediated by NK cells, CD4+ T cells and CD8+ T cells. In acute phase infection, host resistance mainly involves NK cells and CD4+ T cells, and the major IFN-γ producing cells that control parasites in chronically infected mice are CD8+ T cells and to a lesser extent, CD4+ T cells (45). Spleen lymphocytes were re-stimulated with rGRA24, and high levels of spleen lymphocyte proliferation were observed in pVAX1-GRA24-immunized mice. In addition, we measured the proportion of CD4+ T cells and CD8+ T cells in mice immunized with pVAX1-GRA24 by flow cytometry and found that it was significantly higher than in the control groups. Moreover, effective protection against intracellular pathogens depends critically on the induction of cellular immunity, including pathogen-specific CTL responses. However, in many previous articles on *T. gondii* vaccine research, few CTL activities were tested. We examined CTL activity and found that spleen lymphocytes of mice immunized with pVAX1-GRA24 had higher CTL activity than the three controls. These results also indicated that mice immunized with pVAX1-GRA24 had activated special cellular immunity against *T. gondii*.

Mice vaccinated with pVAX1-GRA24 showed a significantly longer survival time than the mice injected with PBS, pVAX1, or nothing, after a lethal challenge with tachyzoites from *T. gondii* RH strain. In the pVAX1-GRA24 immunization group, the average survival time was 24.6 ± 5.5 days, while the mice in the control groups all died by day 7 after infection (*p* < 0.05). However, all mice in our experiment were dead after deadly challenging with *T. gondii* tachyzoites. The current results indicated that the GRA24 can only induce partial protection against infection with high virulent *T. gondii* strain but not complete. However, it is still superior to other single-gene DNA vaccines in terms of survival time. A study showed that a DNA vaccine encoding *T. gondi*i calcium-dependent protein kinase 2 (TgCDPK2) triggered strong cellular and humoral responses and had a prolonged survival time (14.0 ± 2.32 days) in BALB/c mice against a lethal dose challenge of the highly virulent *T. gondii* RH strain compared to control mice (51). Immunization with a DNA vaccine encoding *T. gondii* superoxide dismutase (TgSOD) could trigger strong humoral and cellular immune responses, and had a significant increase in the survival days (median survival of 12.5 days) in comparison to the control groups against acute *T. gondii* ME49 strain infection in the BALB/c mice (52). Another study showed the Kunming mice immunized with pVAX-GRA16 could slightly prolong the survival time (8.4 ± 0.78 days, *p* > 0.05) and significantly decrease brain cysts (43.89%, *p* < 0.05) compared to those in all the controls (53). The survival time in BALB/c mice vaccinated with pSAG4 (9.3 ± 1.64 days) was longer than that of the mice injected by PBS or nothing after virulent *T. gondii* RH strain challenge. All the mice in control groups were dead in 7 days (34). Our previous study found the survival times of mice immunized with pVAX1-TgSPATR were also significantly prolonged (15.7 ± 1.42 days) compared with control groups, which died within 7 days of challenge (*p* < 0.05) (31). Recently, TgROP21 DNA vaccine stimulated potent Th1-type cellular and humoral immunoreaction, prolonged survival time (13.50 ± 1.65 days) after challenge infection with the virulent *T. gondii* RH strain, in comparison to those of control animals (died within 10 days), and decreased the number and size of cysts in the BALB/c mice (35). A growing number of studies have shown that multi-epitope DNA vaccines are more effective against *T. gondii* than single-gene DNA vaccines. Co-administration with pVAX-ROP5 and pVAX-GRA15 boosted the cellular and humoral immune responses, and significantly increased prolonged the survival of immunized mice (22.7 ± 7.2 days) and cyst reduction (79%) (54). Another study showed the survival time of the BALB/c mice (12.3 ± 0.68 days) immunized with pSAG5B/SAG5C was longer than that of the single-gene- immunized mice (8.5 ± 0.53 days) or the control mice (3.8 ± 0.24 days) after a lethal challenge with *T. gondii* RH strain tachyzoites (55). The immunized Kunming mice exhibited longer survival times (44.7 ± 2.1 days for ROP18/PLP1) and lower brain cyst burden (68.9% for ROP18/PLP1) than control mice after *T. gondii* PRU strain challenge (56). By incorporating diverse antigens and an array of adjuvants, DNA vaccines may become more effective against *T. gondii*. In future research, we should evaluate the efficacy and the potential immune mechanism associated with pVAX1-GRA24 immunization by comparing the burden of cysts in the brain tissue of the vaccinated and control groups using strains of *T. gondii* with low virulence, analyzing the potential immune response mechanisms involved and immunizing with other antigens to obtain more effective anti-*T. gondii* results.

In summary, bioinformatic approaches were used to analyze B-cell and T-cell epitopes on GRA24, a novel *T. gondii*. virulence-related protein. In addition, mice were injected with pVAX1-GRA24 DNA vaccines to assess immune protection and the potential immune mechanisms stimulated by the vaccines. Positive humoral and cellular immune responses were stimulated in the mice injected with the DNA vaccine. IRF8, NF-κB, and T-bet pathway activation was analyzed during the host immune response. Vaccination with pVAX1-GRA24 DNA prolonged the survival time of mice challenged with *T. gondii*.

## Ethics Statement

The study was approved by the Animal Care and Use Committee of Zhejiang academy of medical science (Reference No. 2018023), and was performed in compliance with the Regulations for the Administration of Affairs Concerning Experimental Animals, PR, China.

## Author Contributions

BZ, SL, and QK developed the study protocol. BZ, XZ, HD, JD, and DL did the experiments. BZ analyzed the data and wrote the paper. SL revised the report. All authors read and approved the final manuscript.

### Conflict of Interest Statement

The authors declare that the research was conducted in the absence of any commercial or financial relationships that could be construed as a potential conflict of interest.
